# Towards an understanding of tensile deformation in Ti-based bulk metallic glass matrix composites with BCC dendrites

**DOI:** 10.1038/srep22563

**Published:** 2016-03-02

**Authors:** Joanna A Kolodziejska, Henry Kozachkov, Kelly Kranjc, Allen Hunter, Emmanuelle Marquis, William L Johnson, Katharine M Flores, Douglas C Hofmann

**Affiliations:** 1 Keck Laboratory of Engineering, California Institute of Technology, 1200 East California Boulevard, Pasadena, CA 91125, USA; 2 Institute of Materials Science & Engineering, Department of Mechanical Engineering & Materials Science, Washington University, One Brookings Dr., Campus Box 1185, St Louis, MO 63130, USA; 3 Department of Materials Science and Engineering, University of Michigan, Ann Arbor, MI 48109, USA; 4 Materials Development and Manufacturing Technology, Jet Propulsion Laboratory, California Institute of Technology, 4800 Oak Grove Drive, Pasadena, CA 91109, USA

## Abstract

The microstructure and tension ductility of a series of Ti-based bulk metallic glass matrix composite (BMGMC) is investigated by changing content of the β stabilizing element vanadium while holding the volume fraction of dendritic phase constant. The ability to change only one variable in these novel composites has previously been difficult, leading to uninvestigated areas regarding how composition affects properties. It is shown that the tension ductility can range from near zero percent to over ten percent simply by changing the amount of vanadium in the dendritic phase. This approach may prove useful for the future development of these alloys, which have largely been developed experimentally using trial and error.

Bulk metallic glass matrix composites (BMGMCs) have attracted considerable attention due to their exceptional properties, such as their combination of high strength, toughness, and ductility^1^. Different classes of BMGMCs have been investigated, including various *ex-situ* composites, heterostructures^2^, *in-situ* composites with polymorphically crystallized inclusions^3^,^4^, and *in-situ* composites with crystalline dendrites. Of these, *in-situ* Ti and Zr based dendritic composites have demonstrated an exemplary and unique blend of properties and processability. In particular, the composition and volume fraction of the dendrites can be controlled through overall composition^5^, while the morphology of the dendrites can be tailored independently through semi-solid processing, Bridgman solidification and other processing routes. This approach has been used to achieve benchmark ductility and fracture toughness^6^.

Generally, for dendritic BMGMCs to exhibit substantial amounts of tensile ductility and fracture toughness, the dendrites must have a lower modulus than the matrix, such that they inhibit the propagation of shear bands and cracks in the glass matrix^1^,^7^,^8^,^9^,^10^,^11^,^12^. In Ti and Zr based *in-situ* composites, this can be accomplished by the addition of a β stabilizing element, such as vanadium, niobium, or tantalum, to stabilize the dendrites in the high temperature BCC (β) phase. However, since the total concentration of β-stabilizer affects the composition of both the dendrite and the matrix, the dependence of bulk material properties on the concentration of β-stabilizer is not easily quantified.

For the present study, mechanical tensile testing, nanoindentation, atom probe tomography (APT), ultrasonic measurement of sound velocities, XRD, SEM, and TEM microanalysis were used to collect a detailed dataset of the precise compositions and mechanical properties of both phases of a BMGMC system at various bulk β-stabilizer concentrations (in this case, vanadium). To accomplish this, a novel series of BMGMC alloys with β-titanium dendrites was created for this investigation, exploring a broad range of β-stabilizer concentration while maintaining a near-constant dendrite volume fraction, which has previously been extremely difficult to accomplish^13^. To explore the widest possible range of β-stabilizer in this glass forming system, a V-free quaternary composition with sufficient glass forming ability (GFA) was identified: Ti_53_Zr_27_Cu_5_Be_15_, labeled V0. Ti, Zr, and V readily form solid solutions, while Cu and Be have limited solubility in the β phase; thus, the ratio of these two groups of elements essentially determines the dendrite volume fraction of the alloys^5^,^14^. Incrementally increasing the V concentration while holding this ratio constant, the series of compositions is fully defined by Ti_53−x/2_Zr_27−x/2_Cu_5_Be_15_V_x_, where x = 0, 2, 4, 6, 8, and 10 (labeled V0 through V10). DV1 (Ti_48_Zr_20_Cu_5_Be_15_V_12_), a well-studied composite with excellent GFA and mechanical properties, is included as a benchmark, but varies in composition from the series by 1 at.%. Alloys in this series with higher concentrations of V were not investigated due to their limited GFAs.

## Methods

**Figure 1:**
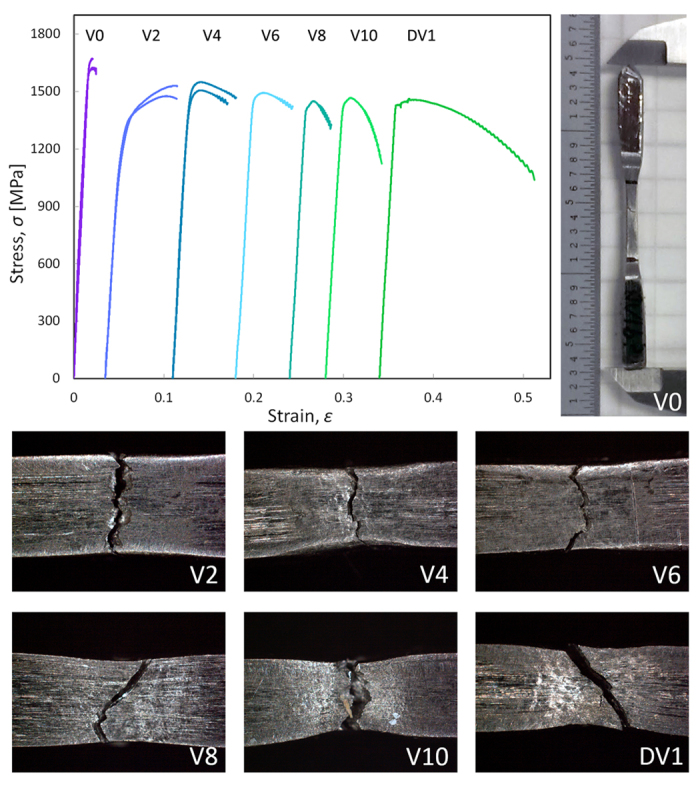
Plot of uniaxial tension test data with optical images of dogbone specimens post-failure. (top left) Engineering stress as a function of engineering strain is plotted from tensile tests on dogbone samples of vanadium series composites. Samples were loaded until failure at a constant strain rate of 0.2 mm/min. Curves are offset on the x-axis to highlight differences in plastic deformation behavior between alloys. (top right) Photograph of complete V0 dogbone sample after failure in tension. (bottom) Optical microscope images at point of failure in deformed alloys V2–V10 and DV1.

Samples of each composition were prepared from >99.9% purity starting materials by arc melting in a Ti-gettered argon atmosphere. The resulting ingots were suction-cast into 4 × 4 mm square cross-section bars using copper molds. XRD was performed on the cast bars using Cu *Kα* radiation on a Philips X-pert Pro to confirm the structure of the BCC dendrites and the amorphous matrix in all bars. The bars were cross-sectioned longitudinally and the center-line microstructure imaged by SEM using a quadrant back scattering detector (QBSD). High-contrast micrographs were segmented programmatically to determine dendrite area fractions (*χ*). The bulk density (*ρ*) of each composite was measured using Archimedes’ method, and speed of sound measurements were collected using 5 MHz transducers for the calculation of bulk elastic moduli: Young’s modulus (E), shear modulus (G), and Poisson’s ratio (*ν*). The indentation hardness (H) and reduced modulus (E_r_) of each phase were measured using a Hysitron TI 950 TriboIndenter with a Berkovich diamond indenter tip at a loading rate of 20 nm/s and 2 s hold at a maximum depth of 100 nm. Results were averaged over 8–12 measurements from different regions of the dendrites, each several indent diameters away from glass/crystalline interfaces, with statistical standard error reported. Due to the presence of Be in the alloy, conventional small-area elemental analysis methods such as energy dispersive spectroscopy (EDS) could not be used accurately. Chemical compositions of each phase were therefore measured using atom probe tomography (APT) analysis. Samples from the matrix and dendrite regions were prepared using *in-situ* liftout within either an FEI 650 Helios Nanolab or Nova 200 Nanolab DualBeam FIB instrument, and final tip sharpening was done with 5 kV Ga ions. APT analysis was performed with a Cameca Local Electrode Atom Probe (LEAP) 4000X HR instrument. Each tip was analyzed using a 355 nm wavelength pulsed laser, with a pulse rate of 250 kHz, incident laser pulse energy of 10.0 pJ, 25 K specimen temperature, and 0.5% average detection rate maintained during acquisition. Tip reconstructions and chemical analysis of each phase were performed using the IVAS software package^15^. The TEM images were obtained with a JEOL 2100 f TEM/STEM (JEOL Ltd. Tokyo, Japan) equipped with a spherical aberration corrector at 200 kV accelerating voltage, in STEM mode with both bright field (BF) and high angle annular dark field (HAADF) detectors. Tension specimens with square gauge sections of 2 mm nominal width and 8 mm gauge length were machined from the cast bars (Fig. 1b) and loaded in tension using an Instron 5969 universal testing machine at a constant displacement rate of 0.2 mm/min until failure. Strain in the gauge section was monitored with a clip-on extensometer. Results are presented in Table 1.

## Results

**Figure 2:**
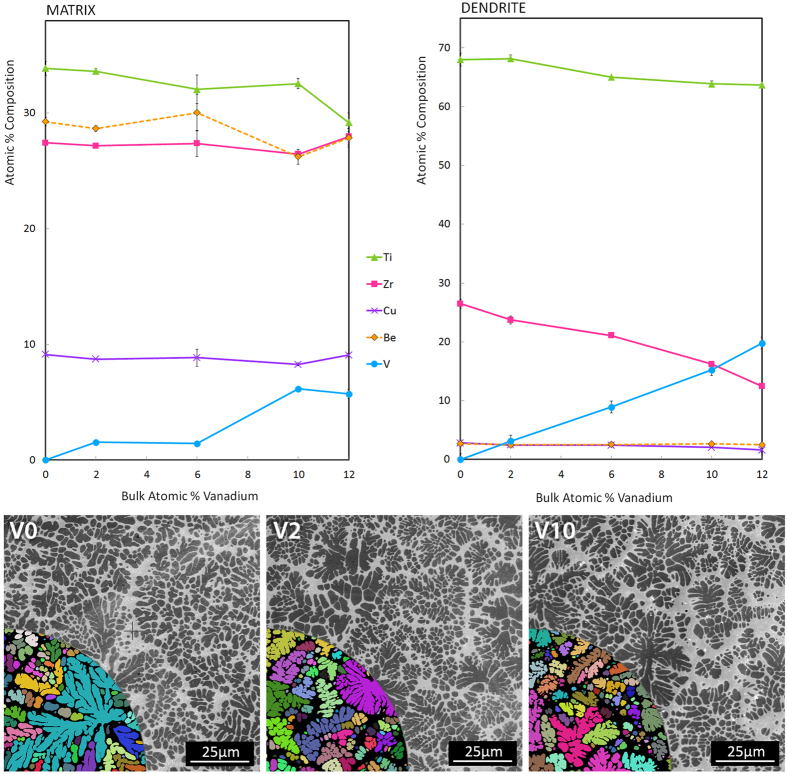
Composition of individual phases obtained from APT; SEM images confirm a relatively constant phase fraction between samples. (top) Elemental composition of the matrix and dendrite phases determined by atom probe tomography and plotted as a function of total vanadium concentration. (bottom) SEM images of V0, V2, and V10, showing similar dendrite morphologies and volume fractions. Colored insets show the results of the segmentation algorithm used to calculate volume fractions.

**Figure 3:**
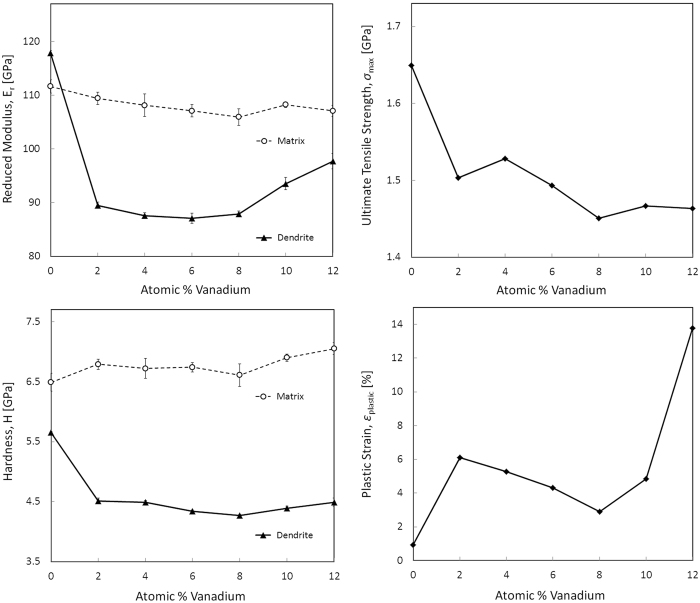
Trends in mechanical properties show local extrema at compositions near V2, and again near V6–V8. (**a**) Reduced modulus (E_r_), (**b**) hardness (H), (**c**) ultimate tensile stress (*σ*_max_), and (**d**) percent plastic strain (*ε*_plastic_) are all plotted as functions of total vanadium concentration. The measurements in (**a**,**b**) were performed using nanoindendation and the measurements in (**c**,**d**) were performed using tension testing.

**Figure 4:**
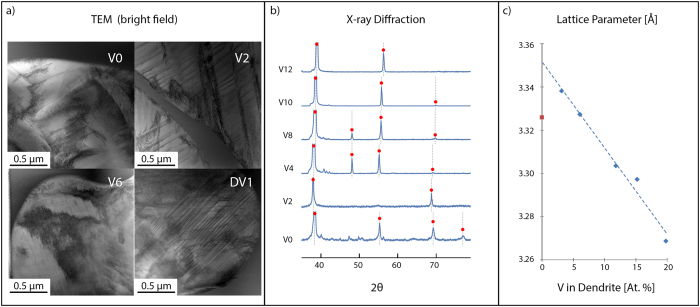
Post-deformation TEM confirms differences in deformation mechanisms; x-ray diffraction analysis suggests the presence of an additional phase in V0. (**a**) TEM micrographs showing the range of deformation mechanisms activated the dendrites of V0, V2, V6, and DV1. Of note, while V2 and DV1 show extensive twinning in addition to dislocations, V6 shows little evidence of twinning, with dislocations as the dominant deformation mechanism. This lack of twinning in the dendrite corresponds to a lack of strain hardening and to a pronounced necking instability in the composite. (**b**) XRD scans confirming the BCC structure of the dendrites. However, the last peak identified in V0 does not correspond to BCC, and provides evidence for the existence of another phase in the V0 dendrite. (**c**) The lattice parameter of the dendrites as a function of vanadium in the dendrite. Blue diamonds correspond to V2, V4, V8, V10, and DV1, while the red square represents V0. The lattice constant of V0 does not fall along the linear fit established by the other compositions providing further evidence of the presence of a third phase.

Figures 1, 2, 3, 4 show the results of the investigation, which include tension tests with micrographs of necking, atomic composition of the matrix and dendrite as measured through APT, SEM micrographs showing the volume fraction of the composites, plots of reduced modulus, tensile strength, hardness and plastic strain as a function of the vanadium content, TEM micrographs showing dislocation damage, and lastly, X-ray diffraction and lattice parameter as a function of vanadium content.

Image analysis of SEM micrographs found similar dendritic microstructures and similar, though not identical, dendrite volume fractions in all the composites. Table 1 shows that dendrite fraction decreased monotonically with vanadium concentration between V0 and a minimum at V8, ranging from 63% to 55% by area, before increasing to 57% for V10. XRD analysis yielded large BCC peaks in all alloys, confirming that each contained β dendrites; V0, uniquely in this series, exhibited an additional clear non-BCC peak. Elastic moduli for the bulk samples obtained through ultrasonic measurement (Table 1) again isolated V0, which exhibited a significantly higher Young’s modulus and shear modulus than the other alloys, as well as a significantly lower Poisson’s ratio. Both moduli dropped to their minima at V2 and then gradually increased for the remainder of the series, except for a slight decrease at V8.

Nanoindentation was used to investigate the mechanical properties of the individual phases. The reduced modulus of the dendrites was drastically higher in V0 than in the other samples, dropping sharply at V2, before dropped slightly but unambiguously between V2 and V4. If any trend exists between V4 and V8, they are obscured, as the moduli of V4, V6, and V8 are not statistically distinguishable. Finally, there was a marked increase in modulus between V8 and V10. Due to smaller statistical uncertainties, the trends in dendrite hardness were less ambiguous. The hardness dropped drastically between V0 and V2, gradually and monotonically decreased between V2 and a minimum at V8, and increased at V10.

To complement the measured mechanical properties of the individual phases, their exact compositions were determined by APT (Fig. 2a,b). These results confirmed the chemical segregation previously measured in similar alloys using EDS, but with greater precision and without the uncertainty stemming from EDS’s inability to detect beryllium. Of particular interest, the dendrites were found to contain ~2.5 at.% Be, which has not yet been reported. The literature has typically set the concentration of Be to zero in the dendrites for phase analysis, due to its low solubility in the BCC phase. Through APT, however, we observe a non-trivial (and near constant) concentration of Be in the dendrites.

Engineering stress-strain curves from tensile tests for all alloys are presented in Fig. 1a. V0 exhibited the highest ultimate tensile stress (*σ*_max_) and limited ductility. In contrast, V2 through V10 all exhibited lower *σ*_max_ and extensive tensile ductility, with varying amounts of strain hardening and necking. Excluding results for V0, *σ*_max_, percent plastic strain (*ε*_plastic_), and the amount of strain hardening - as roughly parameterized by the slope of the stress strain curve after *σ*_max_ -reached a minimum at V8.

Post-deformation TEM micrographs show extensive twinning and dislocations in V2 and DV1, while extensive dislocations dominate in V6. Fig. 4a clearly shows that the dislocation mechanisms vary through the series but without TEM micrographs from V4, V8 and V10, no trends are conclusive. A more detailed TEM study is part of future work.

## Discussion

Figure 1 shows the tension behavior of the series. Two tests were performed on each alloy but the plot only shows the duplicated tests for V0, V2 and V4 due to the unique tensile ductility of those alloys as compared to what is typically observed in these BMGMCs (e.g. DV1). Of particular note is the tensile deformation behavior of V0, which is quaternary BMGMC with composition Ti_53_Zr_27_Cu_5_Be_15_. Based on the reduced modulus and hardness measured by nanoindentation, V0 has dendrites that are stiffer than the matrix, which in the literature has been associated with perfectly brittle behavior similar to a monolithic BMG in tension. It is generally understood that a necessary step towards designing a ductile *in-situ* BMGMC is to ensure that the crystalline dendrites have a lower modulus than the glassy matrix, which V0 does not have, yet in both tests a non-negligible amount of plasticity is observed. TEM micrographs confirm that dislocations were generated in the hard dendrites of V0, even though the sample fails in a globally brittle manner.

The differences in properties and mechanical behavior between V0 and V2 are most drastic in this study. Critically, while the reduced modulus of the matrix remains relatively unchanged at ~100 GPa, the modulus of the crystalline phase falls precipitously (24%) to a value of 89.5 GPa in V2. Changes in the bulk elastic constants of the composite are similarly drastic, with Young’s modulus falling by 22%, shear modulus falling by 24%, and Poisson’s ratio increasing by 11%. Accordingly, V2 exhibits deformation behavior significantly different from that of V0, with extensive tensile ductility, along with strain hardening before failure. In fact, the V2 sample is the only composite in the series to exhibit strain hardening without significant necking. The other samples exhibit ductility that is more typical of BMGMCs: large amounts of highly localized necking with virtually no strain hardening. The transition from the brittle behavior of V0 to the ductile behavior of V2 with only the addition of 2 at.% of vanadium is remarkable and warrants exploration in future studies, particularly by adding vanadium to V0 in smaller increments (say 0.1 at.%). The V2 sample is also interesting from a structural applications perspective, considering that strain hardening is a more attractive mode of tensile failure than pure necking. This behavior has typically only been observed in BMGMCs reinforced with Ta.

The remainder of the alloys in this series exhibit significant tensile ductility with limited strain hardening, as the condition of lower dendrite modulus relative to that of the matrix remains satisfied. However, the extent and character of plastic deformation is not constant, and exhibits trends with composition. In particular, the trends in the tensile behavior of the bulk samples roughly track trends in the mechanical properties of the dendrite determined by nanoindentation. Of note, all trends are reversed at the V6–V8 composition range, where the dendrite modulus, dendrite hardness, *σ*_max_, *ε*_plastic_, and degree of strain hardening all appear to exhibit a local minimum.

Figure 2a,b shows the composition of the matrix and the dendrites of the series as measured through ATP. It should be noted that in the plot V12 is shown even though that alloy has a 1 at.% variance in the amount of titanium and zirconium (as it is the well-studied alloy, DV1). All of the compositions shown in Fig. 2a are bulk glass-forming alloys but with significant differences in GFA. In particular, the quaternary alloy (V0) is a very weak glass former, exhibiting significant crystallization when cast at 5 mm. Figure 4b shows that even when cast into 4 mm beams, some trace of crystallization is still present. The GFA of the quaternary (Ti_53_Zr_27_Cu_5_Be_15_) could easily be improved by increasing the content of Cu, but this alloys series was originally designed for low density^13^, so the Cu and Zr content of the root alloy DV1 was set as low as possible. The rest of the series from V2–V12 are all centimeter or better glass forming alloys but above V12, the GFA rapidly decreases. This is attributed to the increasing V content in the matrix, which is optimal only between 2–8 at.%. Figure 2b shows the composition of the dendrites in the series. The V content in the dendrite increases linearly as the V content in the overall alloy is increased. The increasing V causes a linear decrease in the Ti and Zr content while Cu and Be, which have very low solubility in the Zr-Ti-V BCC phase, remains constant. The micrographs shown in the inset demonstrate that the morphology and volume fraction of the alloys stays virtually constant, which was a requirement for the series to study the effect of the V addition. Traditionally, it has been very difficult to correlate the changes in composition with mechanical properties in BMGMCs because the changes in composition almost always change the volume fraction of dendrites, as well as their properties. By using the formula Ti_53−x/2_Zr_27−x/2_Cu_5_Be_15_V_x_, which sets the amount of Cu and Be at 20 at.% for each alloy, the volume fraction can be maintained. This is due to the low solubility of Cu and Be in the BCC phase and the limited solubility of V in the matrix.

Figure 3 shows trends in the V0–V12 series as measured both microscopically, through nanoindentation, and macroscopically, through tension testing. Figure 3a,b show the reduced modulus and hardness of both the dendrite and the matrix individually. The reduced modulus is a mixed-mode modulus combining both Young’s modulus and shear modulus which is obtained through nanoindentation. For the dendritic phase, both the reduced modulus and the hardness are correlated, showing a very large decrease from V0 to V2. With no V, the modulus of the dendrite in V0 is actually stiffer than the matrix metallic glass, even though the matrix is harder. With the addition of 2 at.% V, both the modulus and the hardness of the dendrite drop dramatically, with virtually no change to the matrix. This is important to note because the significant difference in ductility from V0 to V2 can be attributed to the softening of the dendrite, not to the matrix becoming more brittle. Overall, the modulus of the matrix slightly decreases and the hardness slightly increases from V0–V12. The metallic glass literature has shown strong correlation between lower shear modulus and tougher behavior. As such, the decrease in the reduced modulus from V0–V12 may result in a slight toughening of the glass matrix, which may act to improve the ductility. From V0–V8 the modulus of the dendrite stays relatively constant until it starts to increase at V10. This is most likely attributed to a change in the stability of the β-phase with increasing Ti content, as is seen in crystalline Ti alloys. More analysis of this transition is required, however.

Figure 3c,d show the trends in ultimate tensile strength and plastic strain for the composites as a whole as measured through tension testing. The general trend is that the ultimate tensile strength decreases with increasing V content. The plastic strain shows an initial increase from V0–V2, a decrease from V2–V8 and then an increase from V8–V10. These trends are not necessarily predicable from the nanoindentation measurements on the individual phases. From V2–V8, the reduced modulus of the dendrites stays relatively constant while the matrix decreases. However, the plastic strain from V2–V8 decreases, which is counterintuitive. Moreover, as the dendrites become stiffer and harder from V8–V12, the plastic strain dramatically increases, which is also counterintuitive. The limited TEM micrographs from the deformed samples, shown in Fig. 4a, may offer some clue to this behavior. The deformed V6 sample exhibited extensive dislocation pile-ups, but little to no apparent twinning. In contrast, both V2 and DV1 show extensive twinning in addition to dislocation pile-ups. The inhibition of dislocation motion by twinning or twin boundaries is one of the major mechanisms of strain hardening in β Ti alloys^16^,^17^, and β-Ti BMGMCs^18^,^19^. Although more analysis is needed, the stability of the β phase is the most likely reason for the observed differences in ductility and plastic strain.

Figure 4 shows TEM micrographs of deformed tension specimens, X-ray diffraction data and lattice parameter measurements on the dendrites in the series. The X-ray diffraction data in Fig. 4b shows that the series is all two-phases of BCC dendrites in a glass matrix, with the exception of V0. The last peak in the V0 scan was not identified as BCC and is likely another phase in the dendrite. Elastic stiffening of metastable β Ti alloys is attributed in literature almost entirely to the precipitation of other, stiffer phases^7^. Thus, the significantly elevated modulus of V0 points to the existence of another phase. Furthermore, the calculated lattice parameter for V0, shown in Fig. 4c, deviated from the linear compositional trend of the other alloys, indicative of lattice strain, and further supporting the existence of another phase (Fig. 4). The V0 sample also shows some evidence of crystallization in the matrix, which is attributed to the relatively poor GFA of this alloy. No additional phases were detected by XRD in any of the other alloys, and the relationship between composition and lattice parameter in this region was linear.

In the current work, we have developed a series of BMGMC alloys with a near-constant volume fraction of dendrites, which enabled us to begin to study the effects of an isomorphic beta stabilizer, V, on the mechanical properties of these alloys. The success in developing the new alloy series has opened several new research questions to be explored in future work. In particular, the deformation mechanism in the β Ti dendrites seems to control the mechanical properties of the overall composite. The addition of 2 at.% of V to the quaternary Ti-Zr-Cu-Be BMGMC has a profound effect on the mechanical properties. Additionally, the content of V in the dendrites between 8–12 at.% also has an effect on properties, albeit a less pronounced effect, and the changes are observable through dislocations damage in the dendrites. Overall, this work has shown that 2 at.% V added to a BMGMC results in an alloy with significant work hardening while alloys with 4–12 at.% V exhibit significant ductility but almost entirely through strain softening. The ATP work has shown that the composition of Be in the dendrites is not zero, as previously reported, but is constant at ~2.5 at.%. In summary, this work has shown a significant correlation between composition and mechanical properties of Ti-based BMGMCs, with the potential for tuning the composition to customize performance. This work will hopefully be utilized to develop high-performance structural applications for these unique composites.

## Additional Information

**How to cite this article**: Kolodziejska, J. A. *et al*. Towards an understanding of tensile deformation in Ti-based bulk metallic glass matrix composites with BCC dendrites. *Sci. Rep.* **6**, 22563; doi: 10.1038/srep22563 (2016).

## Figures and Tables

**Table 1:** Experimentally determined properties of bulk metallic glass matrix composites.

*Bulk Alloy Composition*	*Bulk Properties*					*Dendrite Properties*		*Matrix Properties*		*Tensile Test*		
	*χ*	ρ [g/cc]	E [GPa]	G [GPa]	*ν*	E_r,d_ [GPa]	H_d_ [GPa]	E_r,m_ [GPa]	H_m_ [GPa]	σ_max_ [MPa]	*ε*_*tot*_ [%]	*ε*_*plastic*_ [%]
Ti53 Zr27 Cu5 Be15 **V0**	0.63	5.19	107.3	40.5	0.326	117.82 ± 0.47	5.65 ± 0.02	111.63 ± 1.23	6.49 ± 0.15	1649	2.56	0.92
Ti52 Zr26 Cu5 Be15 **V2**	0.61	5.16	83.8	30.8	0.361	89.47 ± 0.59	4.51 ± 0.05	109.41 ± 1.12	6.79 ± 0.09	1503	7.97	6.11
Ti51 Zr25 Cu5 Be15 **V4**	0.59	5.18	85.0	31.2	0.362	87.56 ± 0.54	4.49 ± 0.03	108.13 ± 2.13	6.72 ± 0.17	1528	6.89	5.28
Ti50 Zr24 Cu5 Be15 **V6**	0.56	5.17	88.3	32.4	0.362	87.08 ± 0.93	4.34 ± 0.02	107.08 ± 1.16	6.74 ± 0.08	1493	6.36	4.31
Ti49 Zr23 Cu5 Be15 **V8**	0.55	5.20	87.3	32.1	0.361	87.83 ± 0.56	4.27 ± 0.02	105.92 ± 1.56	6.61 ± 0.19	1450	4.63	2.89
Ti48 Zr22 Cu5 Be15 **V10**	0.57	5.22	90.6	33.4	0.356	93.52 ± 1.16	4.39 ± 0.04	108.24 ± 0.51	6.90 ± 0.06	1467	6.22	4.85
Ti48 Zr20 Cu5 Be15 **V12**	0.58	5.15	94.2	34.4	0.368	97.71 ± 1.41	4.49 ± 0.07	107.07 ± 1.01	7.05 ± 0.10	1477	14.99	13.78

*χ* is the dendrite fraction calculated from SEM images. *ρ* is the bulk density measured using Archimedes’ method. E is Young’s modulus, G is shear modulus, and *ν* is Poisson’s ratio, all calculated from speed of sound measurements on the bulk composite. E_r,d_ and E_r,m_ are the dendrite and matrix reduced moduli, respectively; while H_d_ and H_m_ are the dendrite and matrix hardness values. Both E_r_ and H were determined by nanoindentation, with standard errors reported. *σ*_max_ is the ultimate tensile stress, and *ε*_tot_ is the percent strain to failure from tensile tests on composite dogbone samples. *ε*_plastic_ is the percent plastic strain, calculated by subtracting elastic strain (obtained from extrapolated unloading curves) from *ε*_tot_. Data presented in the bottom row corresponds to benchmark alloy DV1.
